# Modeling of Mechanical Properties of Clay-Reinforced Polymer Nanocomposites Using Deep Neural Network

**DOI:** 10.3390/ma13194266

**Published:** 2020-09-25

**Authors:** Bouchaib Zazoum, Ennouri Triki, Abdel Bachri

**Affiliations:** 1 Department of Mechanical Engineering, Prince Mohammad Bin Fahd University, Al Khobar 31952, Saudi Arabia; 2 CCNB-INNOV, Collège Communautaire du Nouveau-Brunswick, Caraquet, NB E1W 1B6, Canada; ennouri.triki@ccnb.ca; 3 Department of Physics & Engineering, Southern Arkansas University, Magnolia, AR 71753, USA; agbachri@saumag.edu

**Keywords:** polymer, clay, nanocomposites, mechanical properties, deep neural network, back-propagation algorithm

## Abstract

Due to the non-linear characteristics of the processing parameters, predicting the desired properties of nanocomposites using the conventional regression approach is often unsatisfactory. Thus, it is essential to use a machine learning approach to determine the optimum processing parameters. In this study, a backpropagation deep neural network (DNN) with nanoclay and compatibilizer content, and processing parameters as input, was developed to predict the mechanical properties, including tensile modulus and tensile strength, of clay-reinforced polyethylene nanocomposites. The high accuracy of the developed model proves that DNN can be used as an efficient tool for predicting mechanical properties of the nanocomposites in terms of four independent parameters.

## 1. Introduction

Polyethylene is extensively used as an insulated material in electrical and electronic applications due to its high dielectric properties. However, the mechanical properties of this thermoplastic need to be improved. It has been observed that the mechanical properties of nanocomposite materials can be enhanced by adding nanoparticle filler to the polymer matrix [1,2,3,4,5,6,7,8,9,10,11].

Nanoclay materials are considered to be an emerging category of two-dimensional (2D) materials owing to their atomically thin silicate layered structure. The exceptional mechanical properties of nanoclay fillers, namely, high tensile modulus and tensile strength, make them a potential candidate for the enhancement of the mechanical properties of some polymer matrices. This improvement is due to the high contact between the clay platelets and the polymer [12,13,14]. However, the dispersion of the nanoclays into the thermoplastic matrix is a challenge in the manufacturing of nanocomposites, due to the incompatibility of the polymer matrix with nanoclay fillers [15]. Hotta et al. [6] showed that at low nanoclay loading the degree of dispersion has been enhanced, whereas high loading led to difficulty dispersing the nanoclay in the polymer matrix. This is due to the formation of nanoclay aggregates. To improve the dispersion and achieve exfoliation, maleic anhydride modified polyethylene PE-g-MA has been widely used as a compatibilizer for polyolefin-based nanocomposites [16,17].

In previous decades, the deep neural network (DNN) approach has been widely used in many applications, including speech, digit and face recognition; form and object detection; and experiment design. DNN has recently been used as an effective tool to predict the performance of mechanical, electrical and thermal properties of nanocomposites [18,19,20,21,22,23,24,25,26,27,28,29,30]. DNN consists of many small units called neurons, which are grouped into several layer units. DNN takes a number of inputs, carries out numerical processing on those inputs and produces an output.

**Figure 1:**
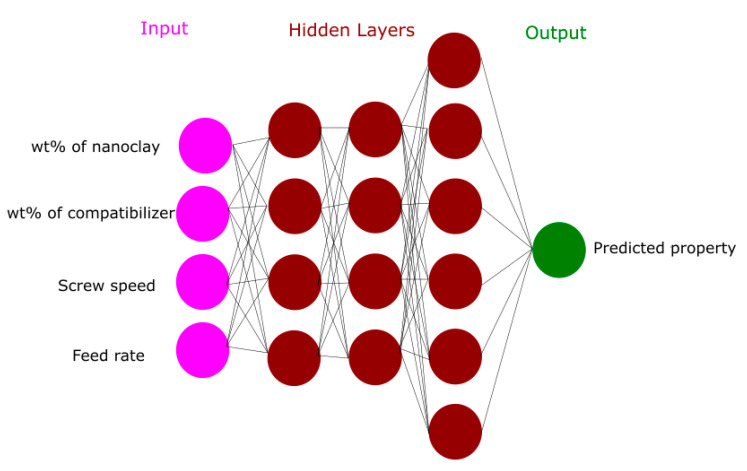
An example of the deep neural network (DNN) structure.

To the extent of our knowledge, there are no results in previous research works on using DNN for modeling the effect of the numerous input parameters, including weight fraction of nanoclay, weight fraction of compatibilizer, screw speed, and feed rate on the mechanical properties of nanoclay-reinforced polyethylene. Figure 1 displays an example of DNN architecture of inputs and outputs.

## 2. Experimental

### 2.1. Materials

Linear low-density polyethylene (LLDPE) with a melt flow index (MFI) of 1 g/10 min was purchased from NOVA Chemicals (Beaver County, PA, USA). A commercially available masterbatch (MB) of LLDPE/nanoclay (NanoMax-LLDEP) containing 50 wt.% of organo-modified montmorillonite (O-MMT) and 50 wt.% of LLDPE, was obtained from Nanocor (Hoffman Estates, IL, USA) and used as a source of the 2D nanoclay fillers. Maleic anhydride-grafted linear low-density polyethylene (LLDPE-g-MA) (Fusabond® M603, DuPont, Wilmington, DE, USA), with MFI of 25 g/10 min was selected as a compatibilizer in this study.

### 2.2. Preparation of Nanocomposites

The MB was further diluted with LLDPE to obtain nanocomposites with different nanoclay content and different processing parameters such as compatibilizer concentration, feed rate and screw speed. All materials were manually pre-mixed before introduction into the twin-screw extruder (Haake Polylab Rheomex OS PTW16, Thermo Fisher, Waltham, MA, USA). The obtained pellets were then press-molded using an electrohydraulic press (178 °C) to form thin plate samples with a thickness of 1.2 mm for mechanical testing.

### 2.3. Characterization

The morphology of the samples was examined using a JEOL JEM-2100F (JEOL, Tokyo, Japan) transmission electron microscope (TEM), with an accelerating voltage of 200 kV. Samples with a thickness of 50–80 nm were cut from the molded plaques of nanocomposites at −120 °C, using a Leica Ultramicrotome (Leica, Germany) equipped with a diamond knife. The tensile tests of LLDPE and its nanocomposites were conducted in accordance with the ASTM D638 standard using the MTS Alliance RF/200 testing machine (MTS, Huntsville, AL, USA) at room temperature with a crosshead speed of 50 mm/min.

## 3. Deep Neural Network

DNN is a computational model inspired by the functional aspects of the human brain. DNN is often used to explore and analyze the correlations between the input and output data sets. Neurons of DNN in each layer receive one input from the neurons of the previous layer and send the output signal to the neurons of the next layer. The main object of DNN is to fine-tune the values of the weights constantly until the predicted data match the target values well. The back-propagation algorithm (BPA) is often used to calculate the error between the target and the predicted data and then to update the weights to diminish this error after appropriate iteration [31].

The output variable *Y* of DNN is given by

(1) $$Y=f\left(\sum_{i}{(W}_{ij}X_{i}{)+b}_{j}\right),$$

where f represents the activation function, $W_{ij}\;$ denotes the weight, $X_{i}$ refers to the *j* the input signal and $b_{j}$ represents the bias. Sigmoid activation function is usually employed as the activation function in the DNN algorithm [32,33,34], which is expressed as follows:

(2) $$f\left(x\right)=\frac{1}{{1+e}^{-\delta x}}\;,$$

where *x* is given by

(3) $$x=\sum_{i}{(X}_{i}W_{i}),$$

and δ  represents the sigmoid function steepness parameter.

Mean Squared Error (*MSE*), Mean Absolute Percentage Error (*MAPE*) and coefficient of correlation *R*^2^ are commonly used for evaluating the accuracy and performance of the DNN model, and they are given as follows:

(4) $$MSE=\frac{1}{N}\sum_{k=1}^{n}{\left(Y_{i}-Y_{k}\right)}^{2},$$

(5) $$MAPE=\frac{1}{N}\sum_{k=1}^{n}(\frac{\left|Y_{i}-Y_{k}\right|}{Y_{i}})\times 100,$$

(6) $$R^{2}=1-\frac{\sum_{k=1}^{n}{\left(Y_{k}-Y_{i}\right)}^{2}}{\sum_{k=1}^{n}{\left(\bar{Y}-Y_{i}\right)}^{2}},$$

where $Y_{i}$ represents the *i*th target value, $Y_{k}$ is the ith predicted value and $\bar{Y_{i}}$ indicates the average of predicted data. *N* is the number of data.

In the present work, Matlab code (Matlab 2014b, The MathWorks, Natick, MA, USA) was written to develop a DNN model of a multi-layer feed-forward network with sigmoid hidden neurons and linear output neurons. The hyperbolic tangent sigmoid function (Equation (2)) is used as the activation function. Weight fraction of nanoclay, weight fraction of compatibilizer, screw speed and feed rate are taken as the inputs and the tensile strength and tensile modulus are the output for the model as described in Figure 1. The backpropagation algorithm is employed for training the neural networks. The Levenberg Marquardt algorithm [35] is used to update the weights and consequently minimize the discrepancy between the output and target values [31]. After several tentative tests, it was found that the optimal DNN architecture that gives the highest correlation coefficient and the lowest relative error has a structure of 4-13-13-12-1, which means four variables in the input layer; three hidden layers with 13, 13 and 12 neurons; and, finally, one predicted output.

In this simulation, 45 data specimens were used; 70% of data were randomly selected for network training, 15% were used to measure network generalization and another 15% were selected for testing. These data have no effect on training and so provide an independent measure of network performance during and after training. Input parameters were nanoclay content (wt.%), screw speed, feed rate and compatibilizer content (wt.%). The predicted parameters were tensile modulus and tensile strength. For each output parameter, a separate neural network has been constructed.

## 4. Results and Discussion

### 4.1. Microstructure Analysis

**Figure 2:**
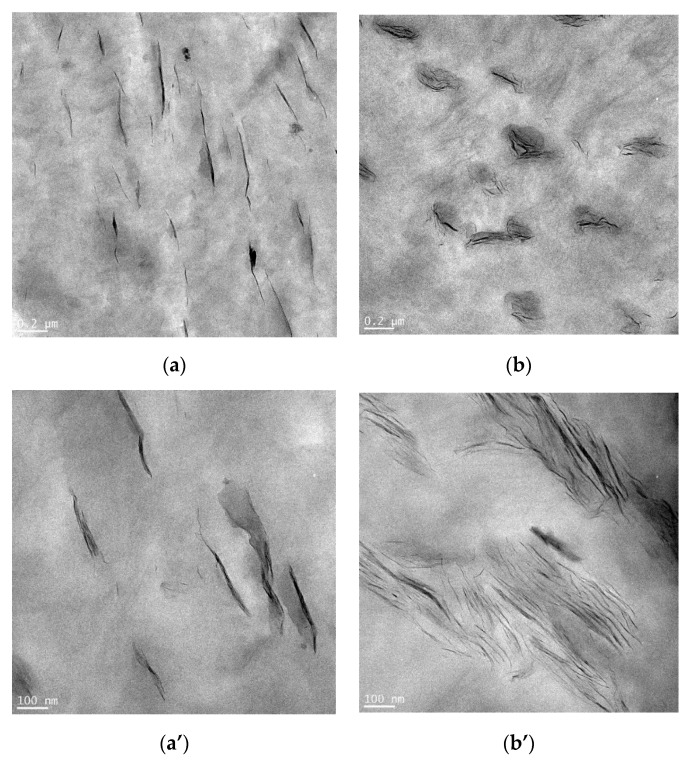
Low and high magnification TEM micrographs for (**a**,**a’**) specimen #4 and (**b**,**b’**) specimen #16.

In the TEM micrographs of specimen #4 and sample #16 nanocomposite materials at low magnification (Figure 2a,b), the two nanocomposites display nearly the same dispersion. At higher magnifications (Figure 2a’,b’), one can better observe the differences in clay dispersion in the polymer matrix. In Figure 2a’, it can be seen that the clay is not well dispersed in the sample #4 nanocomposites. When the compatibilizer (LLDPE-g-MA) was added, the microstructure of the nanocomposites appeared as a combination of intercalated and exfoliated nanoclays, as shown in Figure 2b’. A better dispersion of the clay platelets was achieved for specimen #16 than for the specimen #4 without compatibilizer. This is owing to the polar interactions between the maleic anhydride group of the PE-MA and the OH group of nanoclay [36]. However, Venkatesh et al. showed that nanoclay mixed with compatibilizers (m-TMI-g-PP) is not distributed uniformly and tends to be more aggregated in polypropylene (PP)-nanocomposites than nanocomposites without compatibilizer, wherein a more uniform dispersion of nanoclay is evident [37].

### 4.2. Mechanical Testing

Table 1 displays the effect of the input parameters, namely, clay content, compatibilizer content, screw speed and feed rate, on tensile modulus and tensile strength of the nanocomposites. Results show that reinforcing neat LLDPE with optimum fractions of nanoclay and compatibilizer, and processing ability, results in improved mechanical properties. The variation in tensile modulus and tensile strength indicates the mechanical reinforcing effect of nanoclay in the neat LLDPE. The highest mechanical properties and the better compatibilization efficiency were therefore observed in specific cases. The results are in line with those reported in the literature [37,38]. The change in properties of nanocomposites was also found to be related to the clay and compatibilizer content, and processing parameters. Huitric et al. reported that the elongation at yield and yield strength of LLDPE-nanoclay were improved by the addition of the compatibilizer, whereas the addition of nanoclay was shown to have the opposite effect [38].

**Figure 3:**
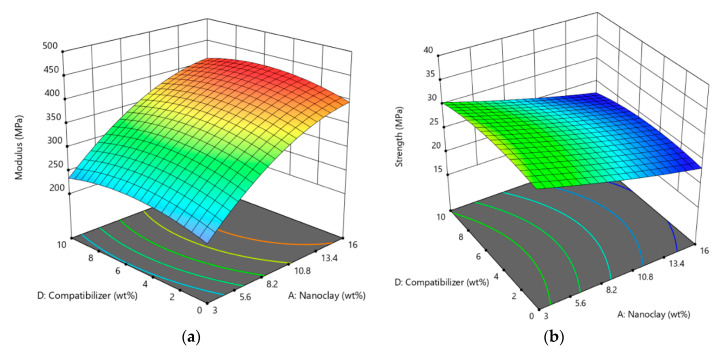
3-Dimensional (3D) surface plot of (**a**) tensile modulus and (**b**) tensile strength versus compatibilizer and nanoclay fractions.

Figure 3 shows the effect of nanoclay and compatibilizer content (wt.%) on tensile modulus (Figure 3a) and tensile strength (Figure 3b) of LLDPE/nanoclay nanocomposites. It can be observed that tensile modulus increased as the nanoclay content increased (Figure 3a). This improvement could be due to the high modulus value of the nanoclay and to the fact that the presence of these nanofillers decreases the mobility of polymer chains resulting in higher tensile modulus [39]. However, the tensile strength decreased as nanoclay content increased, as is shown in Figure 3b. This can be related to the decrease in the degree of dispersion of the nanoclay in the polymer matrix due to the agglomeration. The results also show that the tensile modulus and tensile strength for LLDPE/nanoclay with compatibilizer increased in all nanoclay composition and then decreased. At optimum content of compatibilizer, the tensile modulus and tensile strength of the nanocomposites reached the maximum value, due to the presence of maleic anhydride groups in the LLDPE-g-MA, which facilitated the compatibility between the polymer and the nanoclay, as discussed above. Further increase of the compatibilizer could increase the flexibility of the polymer chains, leading to a decrease of the tensile modulus and tensile strength of the nanocomposites [40].

**Figure 4:**
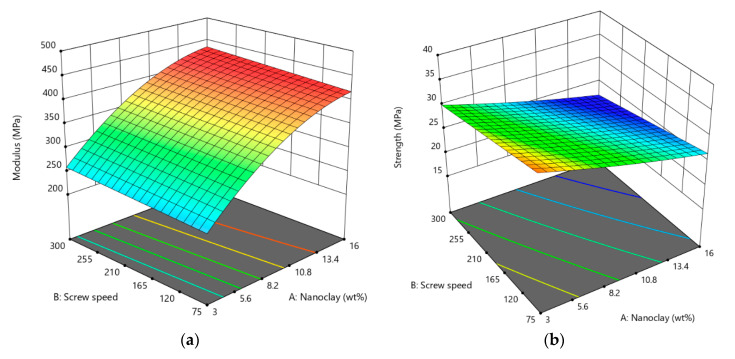
3D surface plot of (**a**) tensile modulus and (**b**) tensile strength versus screw speed and nanoclay content.

As depicted in Figure 4, it is observed that the tensile modulus (Figure 4a) and tensile strength (Figure 4b) slightly increased with an increase in screw speed. This could be due to the fact that high shear stress engendered by screw rotation delaminates clay platelets and improves the quality of dispersion, leading to an enhancement of the mechanical properties. Morever, it can be observed that the value of the tensile modulus was improved with nanoclay loading.

### 4.3. Validation of Neural Networks Model

**Figure 5:**
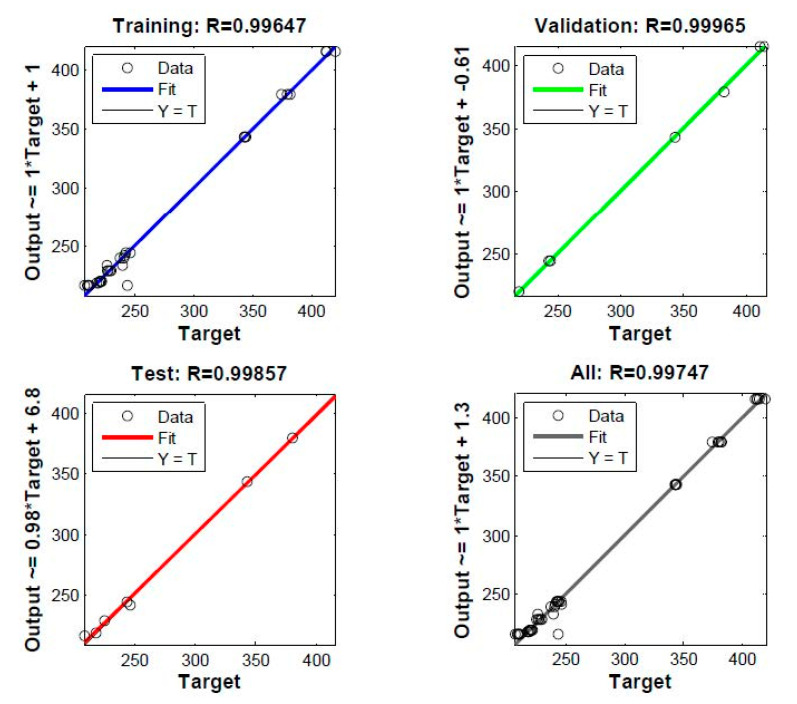
Prediction of modulus by DNN model: regression plot.

**Figure 6:**
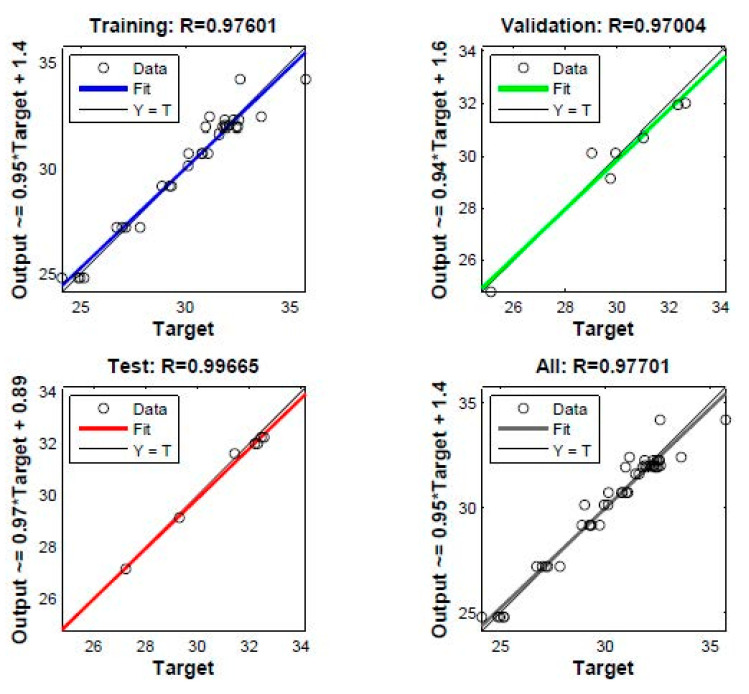
Prediction of strength by DNN model: regression plot.

In constructing the DNN model, the data were divided into three groups: training, validation and testing datasets. The training dataset was used to build the network, the validation dataset was used to validate the models against unseen data and the testing dataset was used to provide an independent measure of network performance. In order to evaluate the validity and accuracy of the proposed DNN model, it is often suitable to do regression analysis between the actual and predicted values. Figure 5 and Figure 6 present the regression analysis for a DNN model with three hidden layers that provided the highest performance for the tensile modulus and tensile strength, respectively. On the top side and y axis of these figures, the coefficients of correlation R and equations of fit that are lined by the format (Output ~ = slope ∗ Target + intercept) are presented for each stage (training, validation and testing). It is clear that the coefficient of correlation R in all stages is close to unity, indicating the validity and accuracy of this DNN model.

Various ranges are collected to ensure the development of a robust model that can be applied to a wide range of the nanocomposites LLDPE-nanoclay. Table 2 and Table 3 show the comparison of actual and predicted values at the testing stage for tensile modulus and tensile strength, respectively. It can be shown that the relative error between experimental results and the predicted data does not exceed 3.60% for the modulus and 1.00% in the case of tensile strength. Thus, this DNN model can predict the tensile modulus and tensile strength of the prepared nanocomposites with satisfactory accuracy.

## 5. Conclusions

In this study, LLDPE/nanoclay nanocomposites were successfully prepared by extrusion process. Mechanical results showed that screw speed and nanoclay loading have a large effect on the tensile modulus, and that tensile strength reached its maximum value at 3 wt.% nanoclay loading, a screw speed of 150 rpm and 2 wt.% compatibilizer content. Additionally, the proposed DNN model presented in this study shows a correlation coefficient value higher than 0.97 during training, validation and test data sets. In addition, the relative error (%) between experimental data and the predicted results does not exceed 3.60% and 1% for tensile modulus and tensile strength, respectively. This confirms the high reliability and accuracy of the proposed DNN model. Moreover, this study proved that DNN can be employed as an efficient tool to predict the satisfactory performance of the mechanical properties of nanocomposites.

## Figures and Tables

**Table 1:** Experimental data for different processing parameters.

Specimen Number	LLDPE Fraction (wt.%)	Nanoclay Fraction (wt.%)	Compatibilizer Fraction (wt.%)	Screw Speed (rpm)	Feed Rate (kg/h)	Tensile Strength (MPa)	Tensile Modulus (MPa)
1	97	3	0	150	0.8	29.94	219.36
2	97	3	0	150	0.8	30.12	218.11
3	97	3	0	150	0.8	29.01	217.67
4	97	3	0	150	1.2	32.34	209.52
5	97	3	0	150	1.2	32.43	211.42
6	97	3	0	150	1.2	31.98	208.74
7	97	3	0	150	1.2	31.78	207.15
8	97	3	0	150	1.2	30.96	210.12
9	97	3	0	150	1.6	32.22	220.34
10	97	3	0	150	1.6	32.10	221.87
11	97	3	0	150	1.6	32.32	219.23
12	97	3	0	150	1.6	32.63	220.91
13	97	3	0	150	1.6	31.89	220.17
14	95	3	2	150	1.2	32.61	246.45
15	92	3	5	150	1.2	31.15	237.27
16	87	3	10	150	1.2	31.43	226.12
17	97	3	0	150	1.2	32.51	243.48
18	95	3	2	150	1.2	35.75	241.15
19	92	3	5	150	1.2	33.63	240.27
20	87	3	10	150	1.2	31.61	239.37
21	95	3	2	75	0.8	32.57	228.48
22	95	3	2	75	0.8	32.30	227.12
23	95	3	2	75	0.8	32.47	225.17
24	95	3	2	75	0.8	31.88	226.72
25	95	3	2	75	0.8	32.56	229.82
26	95	3	2	300	1.6	30.82	243.65
27	95	3	2	300	1.6	31.01	245.87
28	95	3	2	300	1.6	30.78	242.72
29	95	3	2	300	1.6	31.08	244.14
30	95	3	2	300	1.6	30.14	242.12
31	90	8	2	150	1.2	29.23	343.23
32	90	8	2	150	1.2	29.34	344.23
33	90	8	2	150	1.2	29.73	342.89
34	90	8	2	150	1.2	28.87	342.75
35	90	8	2	150	1.2	29.30	343.10
36	86	12	2	150	1.2	27.15	380.26
37	86	12	2	150	1.2	27.24	378.98
38	86	12	2	150	1.2	26.98	382.17
39	86	12	2	150	1.2	27.83	381.42
40	86	12	2	150	1.2	26.72	374.23
41	82	16	2	150	1.2	25.15	412.37
42	82	16	2	150	1.2	25.13	414.23
43	82	16	2	150	1.2	24.97	411.87
44	82	16	2	150	1.2	24.09	420.00
45	82	16	2	150	1.2	24.87	410.72

**Table 2:** Comparison of the experimental and DNN-predicted results of tensile modulus for test samples.

Specimen Number	2	6	14	23	26	33	36
Measured Modulus (MPa)	218.11	208.74	246.45	225.17	243.65	342.89	380.26
Predicted Modulus (MPa)	218.57	216.17	241.61	228.79	244.17	343.33	379.32
Relative Error (%)	0.20	3.60	1.96	1.60	0.21	0.13	0.24

**Table 3:** Comparison of the experimental and DNN-predicted results of tensile strength for test samples.

Specimen Number	9	11	16	21	23	35	37
Measured Strength (MPa)	32.22	32.32	31.43	32.57	32.47	29.3	27.24
Predicted Strength (MPa)	31.99	31.99	31.61	32.24	32.24	29.14	27.16
Relative Error (%)	0.70	1.00	0.57	0.99	0.69	0.52	0.26
